# Recreational football training preserves bone health in women over 9 years during the menopause transition

**DOI:** 10.5114/biolsport.2026.154145

**Published:** 2025-09-09

**Authors:** Magni Mohr, May-Britt Skoradal, Tórur Sjúrðarson, Niklas R. Jørgensen, Jann Mortensen, Peter Krustrup

**Affiliations:** 1 Center of Health Science, Faculty of Health, University of the Faroe Islands, Tórshavn, Faroe Islands; 2 Department of Sports Science and Clinical Biomechanics, SDU Sport and Health Sciences Cluster (SHSC), Faculty of Health Sciences, University of Southern Denmark, Odense, Denmark; 3 Department of Clinical Biochemistry, Copenhagen University Hospital – Rigshospitalet, Copenhagen, Denmark; 4 Faculty of Health and Medical Sciences, University of Copenhagen, Copenhagen, Denmark; 5 Department of Clinical Physiology and Nuclear Medicine, Copenhagen University Hospital – Rigshospitalet, Copenhagen, Denmark; 6 Department of Medicine, The Faroese National Hospital, Torshavn, Faroe Islands; 7 Danish Institute for Advanced Study (DIAS), University of Southern Denmark, Odense, Denmark; 8 Sport and Health Sciences, University of Exeter, Exeter, UK

**Keywords:** Football, Bone mineral density, Bone mineral content, Bone turnover markers, Lean body mass, Physical performance

## Abstract

We examined the long-term effects of recreational football training on bone mineralization, osteogenic activity, and physical function in women during the menopause transition. 25 women (45 ± 4 yrs at recruitment) were randomized into an exercise group (EXE; n = 12) completing 1-h football training sessions on average 1.7/wk for a consecutive 9-yr period or an inactive control group (CON; n = 13). Pre and post, we measured bone mineralization and lean body mass, plasma bone turnover markers, and sprint performance. Significant time × group interactions were for leg BMD, leg BMC, femur shaft BMD, CTX-I, PINP, leg lean mass, and sprint performance (all p < 0.01), favoring the exercise group. Leg bone mineral density (BMD) decreased (P = 0.002) by 0.05 g/cm^2^ [-0.08;-0.02] in CON, but was maintained in EXE, resulting in a higher leg BMD in EXE than CON (P = 0.02). Leg bone mineral content (BMC) increased in EXE (time × group, P = 0.005) resulting in a 56 g [4;108] higher (P = 0.04) total leg BMC in EXE compared to CON. A between-group effect existed in favor of EXE for femur shaft BMD (time × group, P = 0.005). Plasma CTX-I and PINP increased (both P = 0.001) by 95% [43;147] and 64% [29–100] following EXE only (time × group, P = 0.003 and 0.02). Leg lean mass increased (P < 0.001) by 1.5 kg [1.0;2.1] in EXE (time × group, P = 0.006). Finally, sprint performance was maintained in EXE but declined (P < 0.001) by 10% [5;14] in CON (time × group, P = 0.002). Recreational football training (on average 1.7 sessions per week) over 9 years preserves leg bone health, muscle mass and functional capacity in women during the menopause transition.

## INTRODUCTION

Osteoporosis and excessive bone loss are significant global health threats, worsened by demographic changes, with the aging population rapidly increasing [1]. Currently, 200 million people suffer from osteoporosis, a number expected to triple by 2050 [1], with ~9 million fractures annually [2]. Aging leads to reduced bone mass, muscle mass, and physical function, linked to declining sex hormones [3, 4]. Men over 50 and postmenopausal women experience a greater risk of fractures, especially in the lower limbs, hips, and spine [5]. One in three women and one in five men over 50 will experience osteoporotic fractures [6], with postmenopausal women facing increased fracture risk from decreased estrogen production [5, 6], compounded by functional losses like impaired physical function and balance [7].

Exercise has been shown to improve bone health, stimulating osteogenesis [8, 9]. High-impact, high-strain exercise protocols are particularly effective for bone formation [10] and recent studies highlight the effectiveness of hybrid exercise, like football, in promoting bone health [11]. Indeed, the high-impact, multidirectional mechanical loading during football training stimulates bone formation through mechanotransduction [11–13]. Activities like jumping, sprinting, and rapid changes of direction (integral to recreational football) impose forces on the skeleton that can stimulate osteoblast activity and bone remodeling [8–10]. A decade ago, middle-aged Faroese women were enrolled in a trial evaluating the health effects of football and swim interventions [12]. The football group continued for 1 year [13] and has now trained for additional 8 years, providing a unique opportunity to study the long-term effects of hybrid exercise training on bone health during menopause. Thus, the trial represents one of the longest exercise interventions with a control group conducted in women during the menopause transition, where estrogen levels fall, which accelerates bone loss and increases fracture risk [5, 6]. While numerous studies have examined similar, but shorter-duration interventions on bone health in middle-aged populations [11], there is a dearth of data on sustained, long-term exercise adherence and effects. This 9-year follow-up is therefore unique and provides insights not available from previous shorter studies. Finally, we have comparable data on the cohort after 15 weeks [12] and 1 year [13] of training, which also provides a unique long-term overview.

This trial aims to test the hypothesis that long-term and frequent football training can maintain bone health during menopause, focusing on leg BMD and BMC, as well as the femur and spine. Secondary outcomes include the impact on bone turnover markers, and the exploratory hypothesis suggests that hybrid training may help maintain physical function and reduce fall risk.

## MATERIALS AND METHODS

### Participants

A total of 25 women aged 45 ± 4 years at the time of recruitment (baseline) and 54 ± 4 years at follow-up trial participated in this 9-year-long randomized controlled trial (RCT) follow-up. Initially, in 2013, 85 sedentary premenopausal women with moderate hypertension participated. The original cohort was selected among 262 volunteers based on training history, medication, blood pressure and body mass index after detailed screening [12]. 85 participants meet the inclusion criteria, and 44 were randomly assigned to two different swimming groups, and 21 and 20, respectively, were randomly assigned to a hybrid exercise training (recreational football training) group and a control group. The RCT lasted 15 weeks [12] with follow-up after 1 year [13] for only the hybrid training and control groups. The 41 participants that were originally randomized into a hybrid exercise training group (EXE, n = 21) and a control group (CON, n = 20), remained in the same groups and are also part of the present study. Of the 41 initial participants, 25 were included in the present study (EXE: n = 12 and CON: n = 13). In EXE, two women withdrew in the first year (due to time constraints and personal reasons), and an additional 6 withdrew over the subsequent eight years (reasons includes relocation, work/family commitments, and in one case a minor injury unrelated to the study). One exercise participant who remained active in training declined to participate in the 9-year follow-up test. In CON, 7 of the original 20 did not return for the 9-year follow-up (primarily due to loss of interest or inability to be contacted after so many years). There were no differences in baseline characteristics (age, cardiorespiratory fitness, body fat content, blood pressure, leg BMD) of dropouts and the participants completing the 9-year follow-up trial.

The twelve participants in the EXE-group have since March-April 2013 to March-April 2022 participated in regular football-training sessions, while the thirteen participants in the CON-group had not taken up systematic and regular exercise training over the 9-yr long intervention period, which was evident in questionnaires (data not shown). Shortly, the controls completed a questionnaire including the question, “Have you been involved in regular physical training (for months or years) during the last nine years?” Originally, the sample size was chosen based on power calculations of primary outcomes in the original trial, which was blood pressure and body composition, and these data are presented in a recent follow-up paper [14]. We ensured that no participants were on hormone replacement or bone health medications to avoid confounding effects.

### Experimental design

The study is a follow-up randomized controlled trial where the original selection criteria were: a sedentary lifestyle for the last 2 years (no participation in regular training or physical activity); mild hypertension (MAP > 95 mmHg); and body mass index > 25 kg/m^2^. Randomization was done by card drawing first selecting four original groups and thereafter randomizing the groups to different interventions. The original 41 participants were randomized into two groups in 2013, i.e. a football training group conducting exercise training (EXE) and an inactive control group (CON). EXE (n = 12) took part in the long-term recreational football training intervention as well as pre- and post-testing. Throughout the 9-year intervention period, four weekly 1-h training sessions were organized targeting a minimum of 2, but preferably 3 weekly training sessions for each participant. Training attendance was recorded during the entire 9-yr period – during the first year by the supervising coaches and during the last eight years by the participants themselves. The participants in CON did not take part in regular physical training during the 9-yr period, which was shown in questionaries conducted during the follow-up testing. As stated before, from the original EXE-sample 2 participants dropped out during the first year, while 6 others dropped out during the next eight years, while 1 participant, who still is playing, did not volunteer to the 9-yr follow-up testing, resulting in a final sample of 12 participants. The entire original CON-group was invited to take part in the 9-yr follow-up and 13 out of original 20 volunteered. In EXE 11 out of 12 entered the menopauses (age of menopause 49 ± 2 years) during the intervention period, while in CON this was the case in 11 out of 13 participants (age of menopause 50 ± 2 years). Participants had their bone mineral density and content determined, as well as lean body mass assessed, had a fasting blood sample drawn for measurements of bone turnover markers, and performed a 20-m sprint test at baseline and at the 9-year followup time-point. The staff performing the body composition scans and data analysis, as well as blood analysis were blinded from the group identity of the participants. The primary endpoints in the present study were leg, upper femur, and lower spine bone mineralization, as well as bone turnover markers, while the secondary end points were leg lean body mass and 20-m sprint performance.

### Training intervention

EXE performed an average of 1.7 ± 0.6 (± SD) (range: 0.8–2.7) 1-h small-sided football training sessions per week during the 9-yr intervention period, corresponding to a total of 796 ± 275 (range: 374–1264) 1-h small-sided football training sessions in 9 years. Training sessions were organized during the entire year including holiday periods. Four sessions were held each week (three on weekdays and one at the weekend) to ensure flexibility in training attendance. Each session lasted 1 h and consisted of a 10-min warm-up period followed by 4 x 12 min of small-sided football games (4 v 4–8 v 8), as previously described [12]. A trained football coach was present during all training sessions to control the duration of the training, ensure competitive games and record training attendance during the first year of training. After this the participants formed their own football club (FC Trón) and recruited other middle-aged women. During the remaining eight years two participants were made responsible for training every week and training attendance was registered electronically by the responsible participants.

HR and tracking data were only obtained in representative session during the first 15 weeks of the trial and in a session after 9 years of training in order to have an indication of the training load early and late in the intervention. Mean and peak heart rate during a random training session after 9 years were 140 ± 13 and 164 ± 10 bpm corresponding to 81.1 ± 10.1 and 95.7 ± 9.7% HRmax, respectively. Relative heart rate loading was the same as training during the monitored sessions in the initial period of training and in a session 9 years later [14]. On average total distance covered, high intensity running distance and sprinting during training were 4309 ± 636, 220 ± 124 and 51 ± 41 m, respectively, while 571 ± 127 and 608 ± 103 accelerations and decelerations, respectively, were performed per players in the session that was analyzed. Any change in velocity exceeding 1.0 m · s^2^ is defined as an acceleration or deceleration event (this is a common threshold in recreational sports performance analysis) [14].

### Bone mass and lean body mass assessments

Whole-body BMC and areal BMD were evaluated by total body DXA scanning (Norland XR-800, Norland Corporation, USA). The body was segmented in accordance with standard procedures [12, 14] to evaluate regional BMC and BMD, and all analyses were performed using Illuminatus DXA software (Norland Corporation, USA). The leg BMD and BMC were calculated as the average of both legs. We also included separate analyses for the dominant and non-dominant legs based on biomechanical load differences during football training. In addition, a specific region scan was performed of the proximal part of the femur and lower spine. The effective radiation dose was < 0.2 mSv per scan. The DXA scanner used has a test–retest variability (coefficient of variation; CV) of 1.2 and 1.5% for BMD and BMC scans of the femur. In relation to the lumbar spine BMD scans the CV in our laboratory has been shown to be ~1% (based on routine quality control and manufacturer data), and for total-body BMD ~ < 1%. In practice, the least significant change detectable by our DXA for total-body metrics is on the order of 1–2%. The coefficients of variation for repeated measurements in key regions (femur, lumbar spine, total body) are around 1–2%. Whole-body and leg lean body mass was also assessed with DXA scanning, as previously described [15].

### Resting blood sampling

Resting blood sample was collected pre- and post-intervention under standardized conditions from an antecubital vein between 7 and 8 a.m. after an overnight fast using the venipuncture technique. The blood was rapidly centrifuged for 30 s and analyzed by an automatic analyser (Cobas Fara, Roche, France) and the plasma was collected. All fasting blood samples were separated and immediately frozen at -80°C on the day of collection. The plasma samples were frozen and subsequently analysed for BTM, i.e. procollagen type I N propeptide (PINP), osteocalcin, C-terminal telopeptide of type I collagen (CTX-I), using dedicated automated chemiluminescence assays on the iSYS automated analyzer (Immunodiagnostic Systems PLC, Tyne & Wear, UK) at the Department of Clinical Biochemistry, Copenhagen University Hospital Rigshospitalet, Copenhagen, Denmark. The intra-assay CV for these markers is on the order of ~3–5%, and the inter-assay CV is ~5–8%. Specifically, for the IDS-iSYS intact PINP assay, intra-assay CV is ~3–4% and interassay CV ~5%; for CTX-I, intra-assay ~5% and inter-assay ~6%; and for N-MID osteocalcin, intra-assay ~4% and inter-assay ~6–7%. The baseline (Year 0) and follow-up (Year 9) samples were analyzed together in the same analytical run in 2022 to eliminate inter-assay variation over time. Thus, each marker’s change was assessed with identical assay conditions. The samples underwent only one freezethaw cycle (they were thawed once for the final analysis).

### Physical function

Sprint performance was performed as a measurement of physical function using a 20-m sprint test. Sprint time was determined using infrared photoelectric gates with a precision of 0.01 s (NewTest Ltd, Kiviharjuntie, Finland). The participants completed the Yo-Yo intermittent endurance test level 1 (Yo-Yo IE1) as warm-up to the sprint. This type of shuttle-running secures a high muscle temperature [16]. These data are presented elsewhere [14]. A 10-min recovery period separated the Yo-Yo IE1 test and the sprint test. Each participant performed two maximal sprints with strong verbal encouragement separated by 5 min and the best performance was recorded as the test result. The baseline test was performed within 10 days of the first training session, and the 9-yr follow-up test was completed 3–4 days after a training session. The tests were conducted indoors on a wooden surface at environmental temperatures of 18–20°C. All tests were conducted by the same team of investigators using identical equipment and standardized instructions, minimizing inter-individual variability in performance. All participants were given uniform encouragement to ensure maximal performance. The participants were instructed to avoid exercise or intake of alcohol on the day prior to testing and nutritional items rich in caffeine on the day of testing.

### Statistical analysis

In the statistical analysis, we included all participants in the exercise group regardless of their attendance level (an intent-to-treat approach for all who did not drop out). Thus, the results represent the average effect of the exercise program under real-world conditions of adherence. Data are reported as means with 95% confidence intervals (CIs). Primary analyses were conducted using linear mixed-effects models (LMMs) in SPSS (v28, MIXED procedure). Fixed effects included Time (categorical: Year 0, Year 9), Group (exercise, control), and their Group × Time interaction. A random intercept for SubjectID was specified, and repeated measures were modeled at the Time level within SubjectID using an unstructured (UN) covariance matrix. Models were fitted by restricted maximum likelihood (REML) with Kenward–Roger degrees of freedom. Fixed effects were evaluated using Wald F-tests, and planned pairwise comparisons of estimated marginal means (EMMs) were Sidák-adjusted. Baseline values were not entered as covariates; the Group × Time interaction tests whether changes over time differed between groups. Model assumptions were assessed via residual Q–Q plots and residual-versus-fitted plots. Statistical significance was set at P < 0.05.

### Ethics

The study was originally approved by the ethical committee of the Faroe Islands as well as the Sport and Health Sciences Research Ethics Committee at the University of Exeter, Exeter, United Kingdom in 2013, and an extension of the study was approved by the ethical committee of the Faroe Islands in February 2022. The trial was conducted in accordance with the Declaration of Helsinki. After being informed verbally and in writing of the experimental procedures and associated risks, all participants gave their written consent to take part in the study both in 2013 and in 2022.

## RESULTS

### Baseline values

No significant differences existed pre-intervention between the study groups for any of the obtained variables (see Table 1). Furthermore, sub-analysis revealed no differences between completers of the 9-year trial and drop-outs.

**TABLE 1:** Whole-body and regional bone mineral density (BMD) and bone mineral content (BMC) before and after 9 years of either football training or control.

	Outcome	Exercise (n = 11)		Control (n = 12)		P interaction

		Year 0	Year 9	Year 0	Year 9	
Whole-body	BMD (g/cm^2^)	1.03 [0.97;1.10]	1.01 [0.95;1.07]	1.00 [0.94;1.06]	0.94 [0.88;1.00]^***^	0.13
	BMC (g)	2871 [2667;3074]	2775 [2572;2979]	2806 [2612;3001]	2675 [2480;2869]^*^	0.64

Leg	BMD (g/cm^2^)	1.08 [1.02;1.15]	1.08 [1.02;1.15]	1.03 [0.97;1.09]	0.98 [0.92;1.04]^**,#^	0.02
	BMC (g)	504 [466;541]	539 [501;576]^***^	479 [443;515]	483 [447;519]^#^	0.005

Right leg	BMD (g/cm^2^)	1.10 [1.03;1.16]	1.07 [1.01;1.14]	1.04 [0.98;1.10]	0.98 [0.92;1.04]^***, #^	0.09
	BMC (g)	500 [462;537]	532 [494;570]^***^	473 [437;509]	478 [442;514]^#^	0.03

Left leg	BMD (g/cm^2^)	1.07 [1.00;1.14]	1.09 [1.02;1.16]	1.02 [0.96;1.09]	0.98 [0.92;1.05]^*,#^	0.01
	BMC (g)	508 [467;548]	546 [505;586]^**^	486 [447;524]	487 [448;525]^#^	0.02

Femur shaft	BMD (g/cm^2^)	1.06 [0.96;1.15]	1.07 [0.98;1.16]	1.08 [0.99;1.17]	1.02 [0.93;1.11]^**^	0.005
	BMC (g)	16.7 [15.0;18.4]	17.0 [15.3;18.7]	16.7 [15.1;18.3]	15.6 [14.0;17.2]^*^	0.02

Femur neck	BMD (g/cm^2^)	0.91 [0.80;1.02]	0.90 [0.79;1.01]	0.96 [0.86;1.07]	0.84 [0.74;0.95]^**^	0.06
	BMC (g)	3.49 [3.05;3.92]	3.03 [2.60;3.47]	3.15 [2.73;3.56]	2.78 [2.36;3.19]	0.79

Femur trochanter	BMD (g/cm^2^)	0.73 [0.65;0.80]	0.72 [0.65;0.79]	0.73 [0.66;0.80]	0.70 [0.63;0.77]	0.34
	BMC (g)	10.49 [9.26;11.72]	10.67 [9.44;11.90]	10.15 [8.97;11.33]	10.15 [8.98;11.33]	0.70

Femur head	BMD (g/cm^2^)	1.88 [1.69;2.09]	1.87 [1.67;2.04]	1.84 [1.64;2.04]	1.76 [1.56;1.96]	0.44
	BMC (g)	527 [468;586]	514 [455;574]	497 [441;554]	482 [426;539]	0.89

Vert L2	BMD (g/cm^2^)	1.18 [1.05;1.30]	1.09 [0.97;1.22]^*^	1.14 [1.03;1.26]	1.01 [0.89;1.13]^***^	0.28
	BMC (g)	16.92 [14.98;18.85]	16.23 [14.30;18.17]	16.25 [14.40;18.10]	14.18 [12.33;16.03]^**^	0.10

Vert L3	BMD (g/cm^2^)	1.16 [1.04;1.28]	1.11 [0.99;1.23]	1.13 [1.02;1.25]	1.02 [0.90;1.13]^***^	0.15
	BMC (g)	18.52 [16.37;20.67]	17.69 [15.54;19.84]	17.18 [15.12;19.24]	15.84 [13.78;17.90]^*^	0.51

Vert L4	BMD (g/cm^2^)	1.12 [1.01;1.24]	1.11 [0.99;1.22]	1.12 [1.01;1.23]	1.01 [0.90;1.11]^**^	0.07
	BMC (g)	18.81 [16.92;20.70]	19.74 [17.86;21.63]	18.75 [16.94;20.55]	16.42 [14.61;18.22]^**^,^#^	0.005

Values are means with 95% confidence intervals from a linear mixed-model for repeated measures with time, group, and time × group as fixed factors. The P interaction (time × group) value is presented. The result of the post hoc analysis is indicated by

* P < 0.05,

** P < 0.01 and

*** P < 0.001 compared with baseline and

# P < 0.05 compared with football training. The “Leg” is average of right and left leg.

### Bone mineralization and bone mass – 9-year follow-up

#### Leg BMD and BMC

**FIG. 1:**
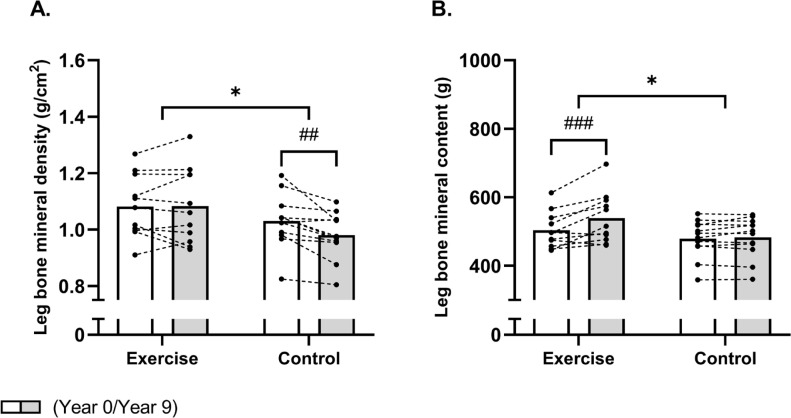
Mean values for leg bone mineral density (A) and content (B) in histograms with individual subjects as lines in exercise (n = 11) and control (n = 12) measured at baseline (year 0; white bars) and following 9 years (grey bars). * Denotes a “time × group” interaction at P < 0.05. # Denotes difference from baseline at P < 0.05. In Figures 1A and 1B, values for the variable Leg are expressed as the average of the right and left leg.

A time × group effect (P interaction = 0.02) existed for leg BMD, which decreased (P = 0.002) by -0.05 [-0.08;-0.02] g/cm^2^ in the control group whereas it was maintained in the EXE, which resulted in a higher leg BMD of 0.10 [0.01;0.19] g/cm^2^ in the EXE compared to CON post-intervention (Table 1, fig 1A). Accordingly, a between-group effect (time × group, P = 0.01) was observed for left leg BMD, which was related to a decreased (P = 0.02) left leg BMD of -0.04 g/cm^2^ [-0.08;-0.01] in the CON group whereas it remained unaltered in the EXE, resulting in a higher (P = 0.03) left leg BMD of 0.11 g/cm^2^ [0.01;0.21] in EXE than CON post-intervention (Table 1). No significant time × group interaction was observed for right leg BMD (P = 0.09). However, post-hoc analysis showed a significant reduction in CON (−0.06 g/cm^2^ [−0.09; −0.03], P < 0.001), resulting in a significantly lower right leg BMD in CON compared to EXE post-intervention (−0.10 g/cm^2^ [−0.18; −0.01], P = 0.04) (Table 1)

With regards to BMC, a between-group effect (time × group, P = 0.005) was also observed for total leg BMC, but this was related to an increased total leg BMC of 35 g [20;51] in EXE as the total leg BMC remained unchanged in CON (Table 1, Fig. 1B). This resulted in a higher (P = 0.04) total leg BMC of 56 g [4;108] in EXE compared to CON post-intervention (Table 1). In accordance, a between-group effect (time × group, P = 0.02) existed for left leg BMC, which was increased (P = 0.002) by 38 g [16;60] following football training and remained unaltered in CON, resulting in a higher (P = 0.04) left leg BMC of 59 g [3;114] in EXE compared to CON post-intervention (Table 1). Furthermore, a betweengroup effect (time × group, P = 0.03) was observed for right leg BMC, which was related to an increased (P < 0.001) right leg BMC of 32 g [15;49] in the EXE only, resulting in a significantly higher (P = 0.04) right leg BMC of 54 g [2;106] following EXE compared to CON post-intervention (Table 1).

#### Femoral BMD and BMC

**FIG. 2:**
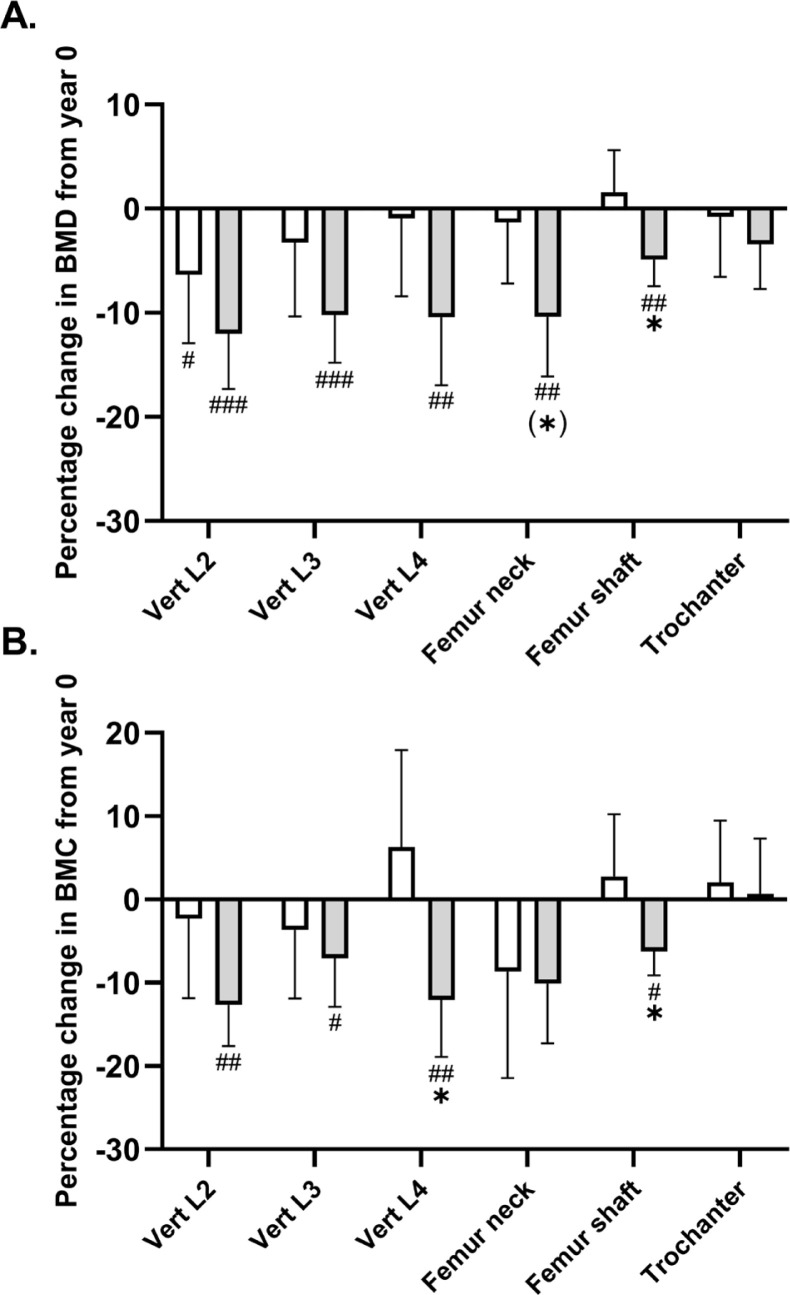
Mean percentage change in bone mineral density (A) and content (B) in the upper part of femur and lower spine in histograms with individual subjects as lines in exercise (n = 11) and control (n = 12) measured at baseline (year 0; white bars) and following 9 years (grey bars). * Denotes a “time × group” interaction at P < 0.05. #,##,### Denotes difference from baseline at P < 0.05, P < 0.01 and P < 0.001.

A significant between-group effect existed in favour of football training for femur shaft BMD (time × group, P = 0.005), with a numeric increase following EXE and a reduction (P = 0.002) of -0.05 g/cm^2^ [-0.09;-0.02] in CON (Table 1, fig 2A). No significant time × group interaction was observed for femur neck BMD (P = 0.06). Post-hoc analysis showed that BMD was maintained in the EXE group (−0.02 g/cm^2^ [−0.09; 0.06], P = 0.70), while a significant decrease of −0.12 g/cm^2^ [−0.20; −0.04] was observed in CON (P = 0.004) (Table 1, Fig. 2A). A similar adaptation pattern was observed for femur shaft BMC (time × group, P = 0.02), which was numerically increased following EXE and reduced (P = 0.01) by -1.1 g [-1.9;-0.2] in CON, but no statistical within-group or between group effect was observed for femoral neck BMC (Table 1, fig. 2B). No statistical between-group or within-group effect existed for femur trochanter or femur head BMD or BMC (Table 1, fig. 2A,B).

#### Lumbar spine BMD and BMC

No significant time × group interaction existed for lumbar vertebrae number 2–4 (L2–L4) BMD (Table 1, Fig. 2A). However, the post-hoc analysis demonstrated a reduced BMD in L2 in both groups, with magnitudes of -0.08 g/cm^2^ [-0.15;-0.01, P = 0.02] in the EXE group and -0.14 [-0.20;-0.07, P < 0.001] g/cm^2^ in CON as compared to baseline. In addition, L3 and L4 BMD was maintained following EXE whereas significant reductions of 0.11 g/cm^2^ [-0.17;-0.05, P < 0.001] and of -0.12 g/cm^2^ [-0.19;-0.04, P = 0.003] existed in CON. With regards to BMC, no statistical between-group effect existed for L2 and L3, but the post-hoc analysis revealed a reduced L2 and L3 BMC of 2.1 g [-3.2;-0.9, P = 0.001] and 1.3 g [-2.5;-0.2, P = 0.02] in CON only (Table 1, fig. 2B). However, a significant between-group effect (time × group, P = 0.005) existed for L4 BMC, which was related to a reduction (P = 0.004) of -2.3 g [-3.8;-0.8] in CON and a non-significant numeric increase of 0.9 g [-0.6;2.5] following EXE, resulting in a statistically higher (P = 0.01) L4 BMC of 3.3 g [0.7;5.9] in EXE compared to CON (Table 1, fig. 2B).

#### Whole-body BMD and BMC

No significant time × group interaction existed for whole-body BMD. However, the post-hoc analysis revealed a reduction (P < 0.001) in whole-body BMD of -0.05 g/cm^2^ [-0.08;-0.03] in CON, while no significant change was detected in the EXE group (-0.03 g/cm^2^ [-0.05;0.00], P = 0.07). Similarly, no significant time × group interaction existed for whole-body BMC, but the pairwise comparison demonstrated a reduced (P = 0.02) whole-body BMC of -132 g [-242;-22] in CON, whereas no statistical changes were observed in the EXE group (-95 g [-210;19], P = 0.10).

### Bone turnover markers, 9-year follow-up

**FIG. 3:**
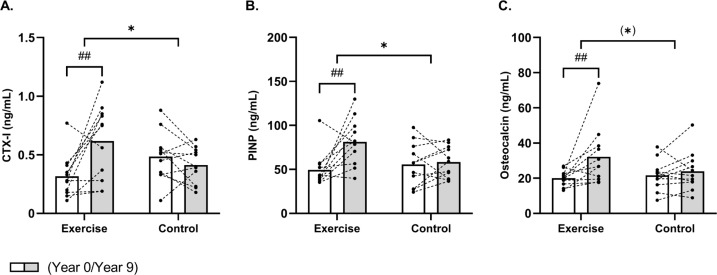
Mean values for bone turnover markers CTX-I (A), PINP (B) and osteocalcin (C) in histograms with individual subjects as lines in exercise (n = 11) and control (n = 12) measured at baseline (year 0; white bars) and following 9 years (grey bars). * Denotes a “time × group” interaction at P < 0.05. ## Denotes difference from baseline at P < 0.01.

A between-group effect (time × group, P = 0.003) existed for CTX-I, which increased (P = 0.001) by 95% [43;147] following EXE only, which resulted in a 49% [2;96] higher (P = 0.04) CTX-I concentration in EXE compared to CON post-intervention (Fig. 3A). Furthermore, a time × group interaction (time × group, P = 0.02) was observed for PINP, which was upregulated (P = 0.001) by 64% [29;100] in the EXE only, resulting in higher (P = 0.02) PINP levels of 39% [6;73] in EXE compared to CON post-intervention (Fig. 3B). No significant time × group interaction was observed for osteocalcin (P = 0.08). However, post-hoc analysis showed a significant increase of 61% [21;100] in the EXE group (P = 0.004), with no change in CON (Fig. 3C).

### Lean body mass and physical function, 9-year follow-up

**FIG. 4:**
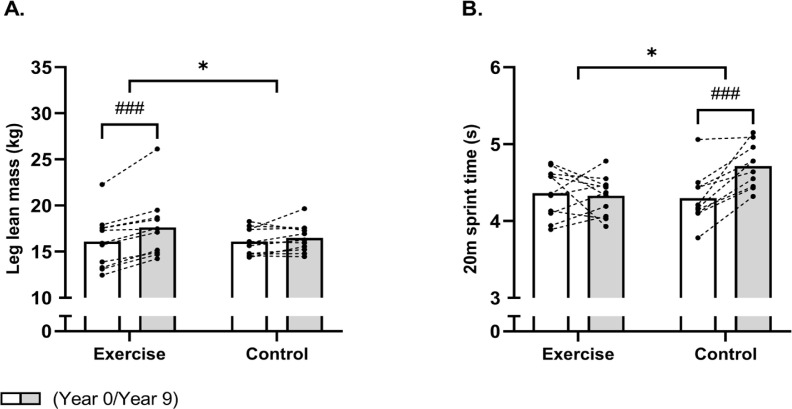
Mean values for leg lean mass (A) and 20-m sprint time (B) in histograms with individual subjects as lines in exercise (n = 11) and control (n = 12) measured at baseline (year 0; white bars) and following 9 years (grey bars). * Denotes a “time × group” interaction at P < 0.05 and parentheses (*) denote a statistical tendency for interaction (P = 0.08). ### Denotes difference from baseline at P < 0.001.

No significant time × group interaction was observed for whole-body lean mass (P = 0.06). Post-hoc analysis showed an increase of 2.8 kg [1.4;4.3] in the EXE group, while no significant change was observed in CON. Furthermore, a time × group interaction (time × group, P = 0.006) was observed for leg lean mass (Fig. 4A). The post-hoc analysis revealed an increased (P < 0.001) leg lean mass of 1.5 kg [1.0;2.1] in EXE, whereas no changes existed in CON.

Finally, a between-group effect (time × group, P = 0.002) also existed in favour of EXE for 20-m sprint time (Fig. 4B). Indeed, the 20-m sprint time was maintained following EXE, whereas it was increased (P < 0.001) by 10% [5;14] in CON, resulting in a faster (P = 0.006) 20-m sprint time of 9% [3;15] in EXE compared to CON post-intervention.

## DISCUSSION

This trial is one of the longest exercise interventions in women during the menopause transition, a critical period for bone health [2, 17]. Our findings show that after 9 years of regular small-sided football training, leg bone mineralization was maintained or improved in the trained group, while sedentary controls saw expected declines. Bone mass at clinically critical sites, such as the proximal femur and lumbar spine, was superior in the trained women. Elevated bone turnover markers further supported the osteogenic effects of long-term multicomponent exercise training. Additionally, the trained women had higher leg lean mass and better physical function than the controls, which may potentially reduce fracture risk.

While no time × group interaction was found at the whole-body level, controls exhibited a significant decline in bone mineral content. In contrast, leg bone mineralization was preserved in the exercise group, with a 35 g increase in leg bone mineral content, supporting earlier studies and meta-analysis evidence on football’s benefits for bone health [11]. Other training studies using football as exercise modality also show positive effects on femur and lumbar spine BMD [18, 19], suggesting that regular multicomponent or hybrid physical activities, like football, effectively preserve bone health during menopause transition. It is interesting that the exercise effects were site-specific: football training predominantly stresses the lower limbs (legs, hips) and to some extent the lumbar spine through weightbearing impact. Consequently, we saw significant preservation in leg BMD (a composite of hip and leg bones) and femoral BMD, whereas the whole-body BMD, which dilutes leg gains with unchanged or naturally declining BMD in other regions, showed only a small net difference. In fact, in our data the exercise group’s whole-body BMD had a slight downward trend (−0.03 g/cm^2^, p = 0.07), whereas the control group had a larger significant drop (−0.05 g/cm^2^, p < 0.001). The interaction was not statistically significant, likely due to insufficient power and the averaging effect. The lack of a significant wholebody BMD interaction effect, despite clear leg-specific benefits, which is in accordance with meta-analysis data [11], can be explained by the inclusion of non-loaded regions in the whole-body measure, which dilutes the robust gains seen at weight-bearing sites. The tracking data also indicate that ~50 m of all-out sprinting and ~220 m of high-intensity running were performed per session (per player), as well as 1200 accelerations and decelerations, defined as change in velocity exceeding 1.0 m · s^2^, were performed in a training session, which are likely to provide a powerful osteogenic stimulus [20]. Thus, this training format inherently includes rapid accelerations, decelerations, cutting maneuvers, jumps, kicking, and intermittent sprinting – all of which impose dynamic loads on the lower extremities, which is paramount for inducing an osteogenic response [10].

Osteoporotic fractures, especially in the hip and spine, are a major health concern worldwide, with one in three women over 50 affected [6]. Our site-specific scans showed preservation of bone mineralization in the femur and lower spine in the trained group, supporting football’s role in maintaining bone health in critical body areas [12, 21]. These findings emphasize the importance of multicomponent exercise training for osteoporosis prevention in women at midlife. The least significant change detectable by our DXA scanner is on the order of 1–2%. Thus, the intervention induced change (e.g., a 5% leg BMD difference) are well exceeding the technical error of measurement, indicating a real effect of the training intervention.

In our trial both groups underwent menopause during the study period, which usually leads to accelerated bone loss due to estrogen deprivation [5, 6]. Thus, the control group’s significant declines in BMD/BMC are consistent with the expected effects of menopause combined with aging and inactivity [5]. In contrast, the exercise group largely maintained bone mass in critical regions despite going through menopause, indicating that regular high-impact exercise can partially counteract or mask the normal osteopenic effect of menopause. This finding is noteworthy given that nearly all participants experienced menopause with its associated drop in estrogen during the trial. Typically, menopause precipitates rapid bone loss; however, the exercise training appears to have mitigated this effect in the exercise group. We acknowledge the lack of measures of hormone levels, and we cannot quantify how each individual’s bone changes correlated with their estrogen status, which should be an aim in future studies to better understand the interaction between exercise and menopause on bone health

Multicomponent training protocols in the form of team sports have proven effective across various populations, upregulating bone turnover markers like CTX-I and PINP [11, 21, 22]. In this trial, the trained group showed significant increases in these markers representing both bone formation (PINP) by 65% and resorption (CTX-1) by 95%. This resulted in higher absolute levels in EXE than CON. Thus, markers of the different phases in bone remodeling coupling were upregulated in the trained group alone, indicating enhanced osteogenesis because of the football training. On the other hand, osteocalcin, representing bone turnover rate, did not reach statistical significance (time × group interaction; P = 0.08), but the post-hoc analysis showed a within-group increase of 61% in the EXE group alone, supporting the hypothesis of elevated osteogenic activity in the football group. This was accompanied by preserved bone mass in key areas, supporting the positive impact and osteogenic dimension of football training, which are further underlined by the tracking data from this and other studies [11].

The benefits of football training were also seen in lean body mass, with the trained women gaining nearly 3 kg in whole-body lean mass, although not statistically significant compared to controls, and 1.5 kg in leg lean mass, which is accordance with meta-data from several population groups after team sports training [23, 24]. The improved muscle mass in the legs is likely to contribute to better functional outcomes, including sprint performance [23]. In line with this, physical function was maintained in the exercise group only despite 9 years of aging, while the controls displayed a 10% decline, consistent with previous studies [25]. Thus, this improvement in leg muscle mass and maintenance of functionality may aid to fall and fracture prevention [10]. It should be mentioned that the exercise training group remained active and the control group generally did not, the motivation factor could potentially play a role. Thus, the longstanding camaraderie in the exercise group likely helped all exercise participants to adhere to the football training session and to put in a comprehensive effort.

Metabolic disorders like poor blood glucose control can negatively affect bone health [26], and football training has been shown to improve blood glucose regulation [20, 23]. Our investigations of the same cohort also found improved cardiometabolic health in the exercise group compared to the controls including a preserved blood glucose regulation [14], suggesting a link between metabolic and bone health. Future studies should explore this relationship further.

In our study, even though not all participants attended the targeted 2–3 sessions per week, huge osteogenic and functional benefits were observed at group level. It is possible that those with higher attendance gained more benefit; however, considering our modest sample we did not perform a subgroup analysis by correlating individual session count with outcome changes to avoid overextending our data. Thus, consistency in training may be equally important and future studies with larger samples should examine dose-response relationships, since there is a lack of long-term such as the present study in the literature. In relation to the control group, we assessed physical activity levels with questionnaires and not objective measurements such as accelerometers. Thus, the lack of rigorous monitoring of the control group’s daily physical activity is a limitation.

Football, as the world’s most popular sport, holds potential as sustainable exercise for preserving bone health throughout life. A feasibility study in the Faroe Islands showed high participation rates, particularly among middle-aged women, supporting the idea that football training is a feasible and motivating way to maintain bone health in women [27]. Recently, a whitepaper was published summarizing existing evidence and suggesting football training as a feasible and sustainable training modality to prevent and treat ten noncommunicable diseases and risk factors across the lifespan with an entire chapter on bone health [28]. Thus, the scalability of the Football Fitness [29] training model appear to be high and can be used be health authorities to preserve bone health in women during the menopause interval. In the present study the dropout rate over 9 years was relatively high (39% overall), however, the reasons appeared unrelated to the intervention itself or the intervention outcomes.

Our participants, while generally healthy aside from mild hypertension, represent a specific demographic subgroup (midlife women in the menopause transition, mostly overweight at baseline). Thus, caution should be in extrapolating the results to other groups, despite that small-sided game football has been successfully tested on several other groups [11, 18, 23]. The study is the first to look at this type of intervention in peri- to postmenopausal women over such a long period, and while the results are promising for this group, further research would be needed to confirm similar benefits in other populations.

While the study provides valuable insights, especially in relation to the long duration of the trial, it has limitations, including a small sample size, potential bias from participant attrition, and lack of comprehensive data on participants’ overall physical activity, hormonal status and diet. Especially, the absence of physical activity, hormonal, vitamin D status and nutritional data means we cannot ascertain their influence on bone outcomes in the present study. However, despite the lack of direct hormonal measurements, the two groups were similar in menopausal timing, and all participants were free of hormone replacement therapy or bone-affecting medications (see Methods). Future studies should address these limitations and further explore daily physical activity, hormonal influences and dietary factors on bone health. Another limitation may be that attrition could potentially introduce bias, even though those who remained were not evidently different from those who left. Finally, the limited sample size and consequent limited statistical power as a key limitation. Especially, there may be a risk of a statistical type 2 error, and that it may be hard to reach significance for key variables due to insufficient power. However, despite the small sample size, several key outcomes did show statistically significant group differences (e.g., leg BMC, leg BMD, femoral shaft BMD, leg lean mass, sprint performance), indicating a robust signal given the magnitude of changes over 9 years. Also, consistently, almost all exercise participants had either maintenance or improvement of leg BMD over baseline, whereas most controls had declines, as seen from the figure with individual as well as average values for the two groups. Finally, lack of rigorous monitoring of the control group’s lifestyle is a limitation.

## CONCLUSIONS

The present study revealed that nine years of regular recreational football training, performed 1.7 times per week on average preserved leg BMD and BMC, increased leg muscle mass, and maintained physical performance in middle-aged women during the menopause transition, compared to the age-related declines in a non-training control group.
